# Taste Preferences in Broilers: Behavioral Evaluation for Varying Concentrations of Four Essential Amino Acids

**DOI:** 10.3390/ani15111574

**Published:** 2025-05-28

**Authors:** Jaime Figueroa, Paloma Cordero, Sofía Herrera-Alcaíno, Sergio A. Guzmán-Pino

**Affiliations:** 1 Instituto de Ciencias Agroalimentarias, Animales y Ambientales—ICA3, Universidad de O’Higgins, San Fernando 3070000, Chile; jaime.figueroa@uoh.cl; 2 Programa de Doctorado en Ciencias Silvoagropecuarias y Veterinarias, Campus Sur, Universidad de Chile, Santiago 8820808, Chile; paloma.cordero@veterinaria.uchile.cl (P.C.); sofia.herrera.a@ug.uchile.cl (S.H.-A.); 3 Departamento de Fomento de la Producción Animal, Facultad de Ciencias Veterinarias y Pecuarias, Universidad de Chile, Santa Rosa 11735, La Pintana, Santiago 8820000, Chile

**Keywords:** acceptability, broiler chicken, essential amino acid, peak cluster size, preference test, sensory-motivated intake, umami taste

## Abstract

Umami taste compounds are perceived in broilers through taste buds that detect peptides and amino acids, which can positively or negatively affect their feeding behavior. In this study, we evaluated the intake behavior for four essential amino acids (Lysine, Methionine, Threonine, and Tryptophan) in chickens. Sixty-four one-day-old male birds (Ross 308) were used. For 16 days during the early stage of the birds, two-choice preference tests were performed, in which 16 combinations composed of four amino acids in four concentrations (0.1 to 1.5%) diluted in water were evaluated, which were supplied in contrast to the delivery of water (a neutral compound) to a pair of birds in a pen for 4 h of administration after a prior 1 h fast. Amino acid solutions such as Threonine and Tryptophan tended to show less preference at the highest exposed concentrations (1.5%) concerning drinking water, which was confirmed in the case of Threonine when performing a sensory-motivated intake analysis (SMI). The opposite occurred with Lysine (1.5%), which numerically showed a higher preference ratified by SMI and acceptability analysis concerning water and other concentrations of the same amino acid, respectively. When palatability was measured with pecking cluster size, no significant differences across amino acid concentrations were observed, which is probably attributed to short recording periods and differences in solution intake behavior between chickens and previous experimental models such as rats. The results reinforce the notion that it is necessary to standardize feeding behavior tests in birds according to their feeding patterns and nutritional needs.

## Simple Summary

This study examined how chickens respond to essential amino acid solutions. After a one-hour fast, we tested 64 one-day-old male chickens over 16 days, offering them Lysine, Methionine, Threonine, and Tryptophan in concentrations ranging from 0.1% to 1.5% through two-choice preference tests. Amino acid solutions such as Threonine and Tryptophan at 1.5% tended to show less preference than drinking water, which was confirmed in the case of Threonine when performing a sensory-motivated intake (SMI) analysis. However, Lysine (1.5%) numerically showed a higher preference ratified by SMI and acceptability analysis concerning water and other concentrations of the same amino acid, respectively. No palatability differences across amino acid concentrations were observed, which is probably attributed to differences in solution intake behavior between chickens and other animal models such as rats. These findings support the idea that it is essential to standardize feeding behavior assessments in birds based on their feeding patterns and nutritional requirements.

## 1. Introduction

Food choice and animal feed consumption depend primarily on odor and taste perception [1]. Taste buds are the peripheral sensory organs of taste and are essential in guiding animals to select and prioritize nutrients. Previous studies indicate that avian species, such as broiler chickens, have a well-developed sense of taste and are able to distinguish at least five primary tastes [1,2,3]. Among them, the umami taste is mainly related to the protein fraction of the diet and the detection of some L-amino acids, peptides, and other products related to protein degradation [4,5]. These compounds can influence the intake and palatability of the feed offered to chickens [6], allowing producers to improve the poultry industry’s efficiency by adding different components to the animals’ diet that can increase motivation for consumption and improve production parameters [5,6].

Because productive animals do not have the opportunity to express consumption preferences for different ingredients like humans, it is necessary to perform feeding behavior assays to study their perception and palatability of sapid compounds [5]. Preference (choice) tests that measure the intake of each compound offered are commonly used for this type of analysis [5]. Although umami has been considered an attractive taste, some studies indicate that broiler chickens may show a weak preference for umami taste solutions or even an aversion [1,2]. To date, preference studies in broilers have been dedicated to evaluating the use of umami compounds and/or their flavor enhancers. For example, in a recent study from our group, we determined that broiler chickens displayed changes in their preferences when evaluating compounds at different concentrations and consumption measurement times, resulting in significant preferences for monosodium glutamate (MSG) 150 mM at 4 h and 8 h and 300 mM MSG at 2 h when evaluated in pairs of birds during days 7–23 of the productive cycle [7]. However, no studies currently exist that analyze chickens’ preferences for essential amino acids typically included in diets with growing purposes.

It is also important to mention that studies on umami taste perception in poultry have been limited only to preference trials without evaluating the acceptability or palatability of the amino acids or compounds given. Palatability is the hedonic reward provided by foods or fluids pleasing to the palate concerning the homeostatic satisfaction of water or nutritional needs [8]. In general, methods used to assess animal palatability typically estimate feeding behavior components in preference and/or acceptability tests [9]. However, preference tests are limited by the possibility of an interaction between the options offered and the exposed time. In the case of acceptability tests, studies in other species have shown that high concentrations of compounds can promote satiety and a reduction in consumption without necessarily decreasing palatability. Therefore, a preference or higher consumption of a solution or food does not necessarily mean it is more palatable [10]. The lick cluster size method, used in rats, consists of quantifying the number of licks that the animal performs in a given number of approaches to the solution where the cluster size increases compared to more palatable solutions that are associated with a greater presence of nutrients [10]. In birds, in the absence of licking behavior, it would be possible to extrapolate the number of licks and the number of approaches to the number of pecks and number of bouts, respectively, according to previously described parameters [7,11]. In this way, it would be possible to describe a consumption pattern or peak cluster size similar to that of other species that generates better approximations of the palatability of amino acids. Thus, this work aimed to evaluate broiler chickens’ preference thresholds, sensory-motivated intake (SMI), acceptability, and peak cluster size for Lysine, Methionine, Threonine, and Tryptophan to provide a better understanding of the intake behaviors for amino acids.

## 2. Materials and Methods

### 2.1. Animals, Housing, and Diets

Following the methodological recommendations previously published [7], 64 early-stage male broiler chickens (Ross 308) were distributed in pairs in the Experimental Unit for Poultry Nutrition and Production of the Faculty of Veterinary and Animal Sciences of the University of Chile. This facility features a traditional design with natural airflow and 32-floor pens equipped with wood-shaving bedding. It is warmed by gas brooders that include temperature control, and it has automatic drinkers and individual feeders positioned within each pen. The environmental parameters for temperature and relative humidity schedules were established following the guidelines of the genetic strain, and lighting was implemented with 23 h of light and 1 h of darkness from 0 to 7 days and 19 h of light and 5 h of darkness from 8 to 23 days according to the breeder’s recommendations [12]. Animals were fed a starter commercial diet (Supplementary Table S1; PROA S.A., Santiago, Chile) formulated to fulfill all nutritional requirements set by the guidelines provided by the breeder [13]. The diet was made available ad libitum to the chickens during the entire assay period, and water was also offered ad libitum until the preference tests were initiated.

### 2.2. Experimental Design

**Figure 1:**
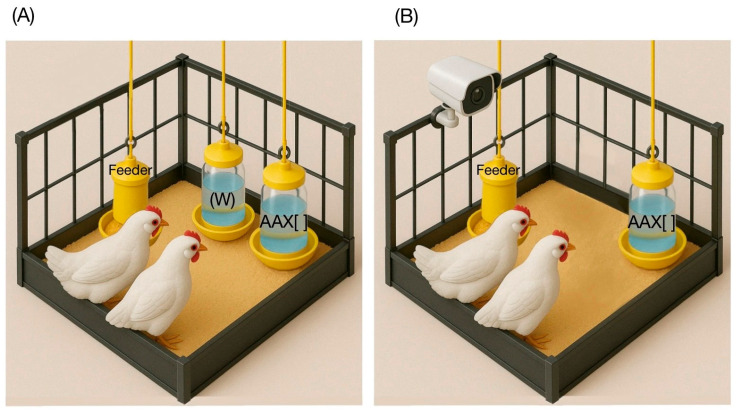
Scheme of experimental methodology. (**A**) An example of the delivery of “W” and “AA × []” matrixes in a pen for preference/SMI tests. (**B**) An example of delivering a single “AA × []” matrix in a pen for acceptability/pecking cluster size tests.

Upon arrival, the one-day-old birds were subjected to an initial phase of 7 days of acclimatization to the environmental conditions of the Poultry Unit prior to the start of the experimental tests on day 8 of life. The synthetic essential amino acids Lysine, Methionine, Threonine, and Tryptophan (purity > 99%; Veterquímica S.A., Santiago, Chile) were evaluated at 0.1%, 0.5%, 1%, and 1.5% concentrations. Delivery was carried out rotating for 16 days, determining that all amino acids were tested in all concentrations counterbalanced in each pen. Every test day started at 8:00 am with a 60 min water fasting period for the birds. The 32 pens were randomly divided into two groups and assigned to perform preference/SMI tests or acceptability/pecking cluster size tests. In the case of the former, at 9:00 am, two identical drinkers were positioned 20 cm apart (Figure 1A), with one containing plain drinking water (“W”) and the other containing an amino acid diluted in water at a specific concentration (amino acid × concentration, “AA × []”), following the previous literature [2,7,14,15]. Drinkers’ right/left placement was alternated daily to prevent preference biases related to the birds’ habituation. The second case offered a single drinker with a specific AA × [] combination (Figure 1B). For both methodologies, the test lasted 4 h each day. The weights of all drinkers were recorded before they were placed in the pens and again when they were taken out, allowing for the calculation of consumption by subtracting the amount remaining from the amount offered. The birds’ consumption was assessed based on their metabolic weight during the testing period, considering their age-related differences in body weight (BW). This was expressed in g/kg of BW and utilized to determine differential solution intakes.

#### 2.2.1. Preference Thresholds

Preference was quantified as a percentage ranging from 0 to 100%, with 50% representing a zone of indifference or neutrality. Values exceeding 50% indicated a zone of preference. The preference value was calculated by assessing the percentage of consumption of AA × [] relative to the total intake (which includes both AA × [] consumption and W consumption), measured against the neutral benchmark of 50%. This is articulated through the following formula:

$$Preference\left(\%\right)=\frac{AA\times []\;consumption}{AA\times []\;consumption+W\;consumption}\times 100$$

#### 2.2.2. Sensory-Motivated Intake

Sensory-motivated intake serves as a valuable metric, offering more profound insights into how the consumption of a specific AA × [] combination increases when positively influenced [4]. This parameter enhances our understanding of preference measurements. The calculation involved determining the difference between the consumption of the administered solutions and non-consumption, as illustrated in the following formula:

$$SMI\left(g\right)=AA\times []\;consumption-W\;consumption$$

A positive SMI value indicated that the AA × [] consumption surpassed that of the neutral compound. In contrast, a negative SMI value implied that the AA × [] consumption fell short compared to the water.

#### 2.2.3. Acceptability

In each pen, a single drinker was provided with the AA × [] combination solution for 4 h. The difference between the weight of the solution offered at the beginning and the weight of the solution recovered at the end of the test was regarded as the consumption or solution acceptability of the animals:

$$Acceptability\left(g\right)=AA\times []\;consumption$$

#### 2.2.4. Pecking Cluster Size

Broiler chickens were monitored during the first 10 min of acceptability tests using cameras Ezviz^®^ IP WIFI 2MP (Hangzhou, China) positioned above the pen. This setup provided an optimal viewing angle for capturing behavioral observations while the chickens were exposed to the solutions. The number of pecking bouts was recorded [11]. Subsequently, the consumption pattern, or pecking cluster size, was calculated to estimate the palatability of the amino acids in this study using the following formula:

$$Pecking\;cluster\;size=\frac{Number\;of\;pecks}{Number\;of\;bouts}$$

### 2.3. Statistical Analysis

The normality and homogeneity of variance for each variable were assessed using the Shapiro–Wilk and Levene’s tests. The mean preference and SMI values were compared to the neutral preference (50%) and the negative control (0 g). These data were analyzed using Student’s *t*-tests through the MEANS procedure in SAS (version 9.4, SAS Institute; Cary, NC, USA). The acceptability and pecking cluster size of amino acids were examined using a two-way ANOVA followed by Tukey’s post hoc multiple comparisons, utilizing the GLM procedure in SAS. This analysis considered the effects of each amino acid, its concentration, and its interaction as primary factors. Each pair of animals was treated as the experimental unit in all analyses. A significance level of α = 0.05 was applied, and values between 0.050 < *p* < 0.100 were interpreted as trends.

## 3. Results

### 3.1. Two-Choice Preference Tests

**Figure 2:**
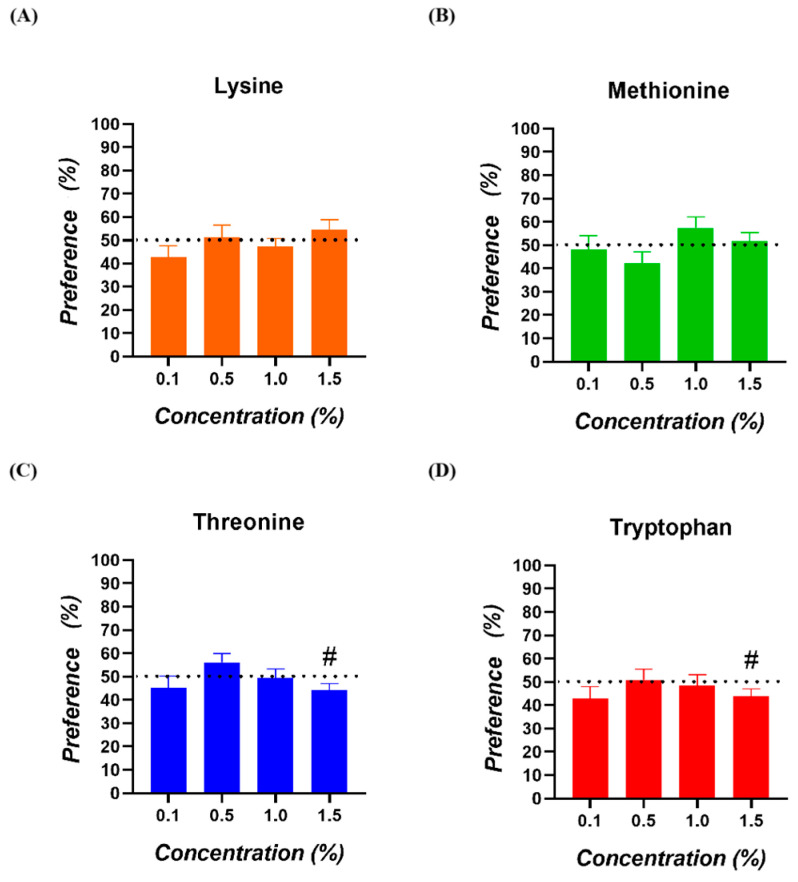
Preference of broiler chickens for (**A**) Lysine, (**B**) Methionine, (**C**) Threonine, and (**D**) Tryptophan. The dotted line (50%) indicates the neutral or lack of preference zone. (#) = values with a tendency towards significance (0.050 < *p* < 0.100).

No significant preferences for any of the amino acids and concentrations tested were observed (*p* > 0.050; Figure 2). In the case of Threonine and Tryptophan at 1.5%, a tendency for a lower preference was registered (*p* = 0.063 and *p* = 0.079, respectively).

### 3.2. Sensory-Motivated Intake

**Figure 3:**
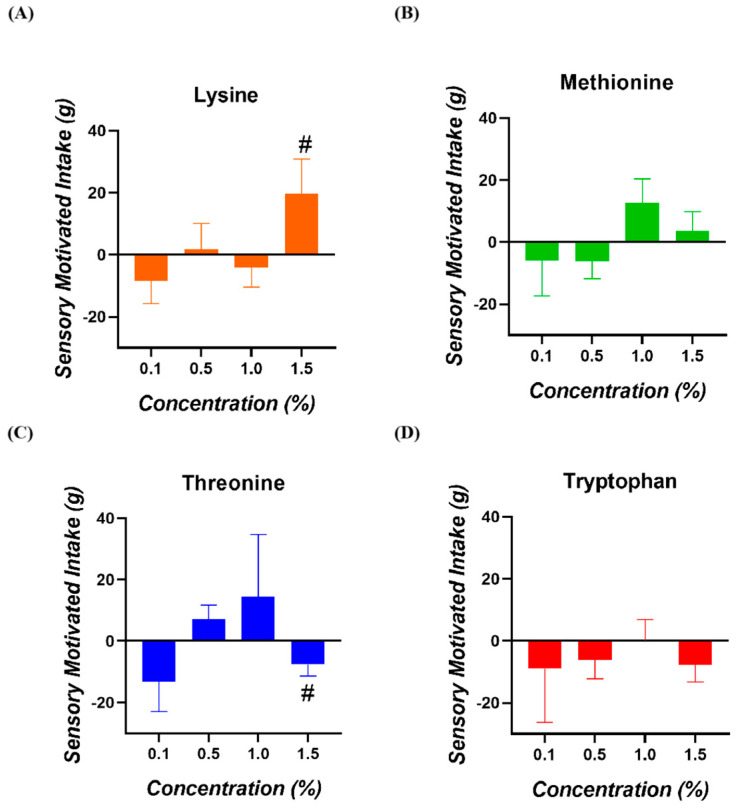
Sensory-motivated intake of broiler chickens for (**A**) Lysine, (**B**) Methionine, (**C**) Threonine, and (**D**) Tryptophan. (#) = values with a tendency towards significance (0.050 < *p* < 0.100).

No significant SMI values for any of the amino acids and concentrations tested were observed (*p* > 0.050; Figure 3). In the case of Lysine at 1.5%, a tendency for a higher SMI was registered (*p* = 0.098). Concerning Threonine at 1.5%, a tendency for a lower SMI was registered (*p* = 0.068).

### 3.3. Acceptability

**Figure 4:**
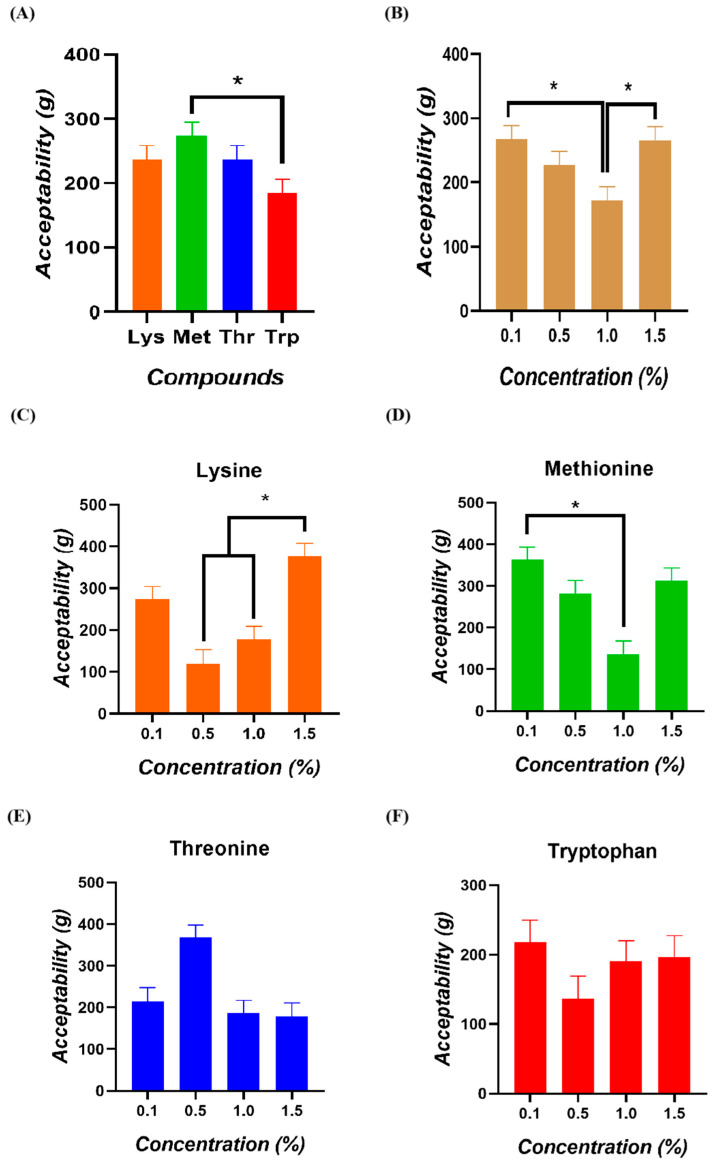
Acceptability of broiler chickens (**A**) among four amino acids, (**B**) four concentrations, and for (**C**) Lysine, (**D**) Methionine, (**E**) Threonine, and (**F**) Tryptophan. (*) = consumption values significantly different (*p* < 0.050).

The comparative analysis of consumption among amino acids determined a higher intake of Methionine compared to Tryptophan (*p* = 0.033; Figure 4A). When analyzing acceptability according to the concentration of the amino acids delivered, higher total intakes of all compounds were determined at 0.1% and 1.5% compared to the 1.0% concentration (*p* = 0.004; Figure 4B). When determining the acceptability of each compound according to the delivered concentrations, significantly higher mean intakes of Lysine were observed at the 1.5% concentration compared to the 0.5 and 1% concentrations (*p* < 0.050; Figure 4C). For Methionine, significantly higher mean intakes were observed at the 0.1% concentration than at the 1.0% concentration (*p* < 0.001; Figure 4D). For Threonine and Tryptophan, Tukey’s post hoc analysis did not reveal significant differences between concentrations (Figure 4E,F).

### 3.4. Pecking Cluster Size

**Figure 5:**
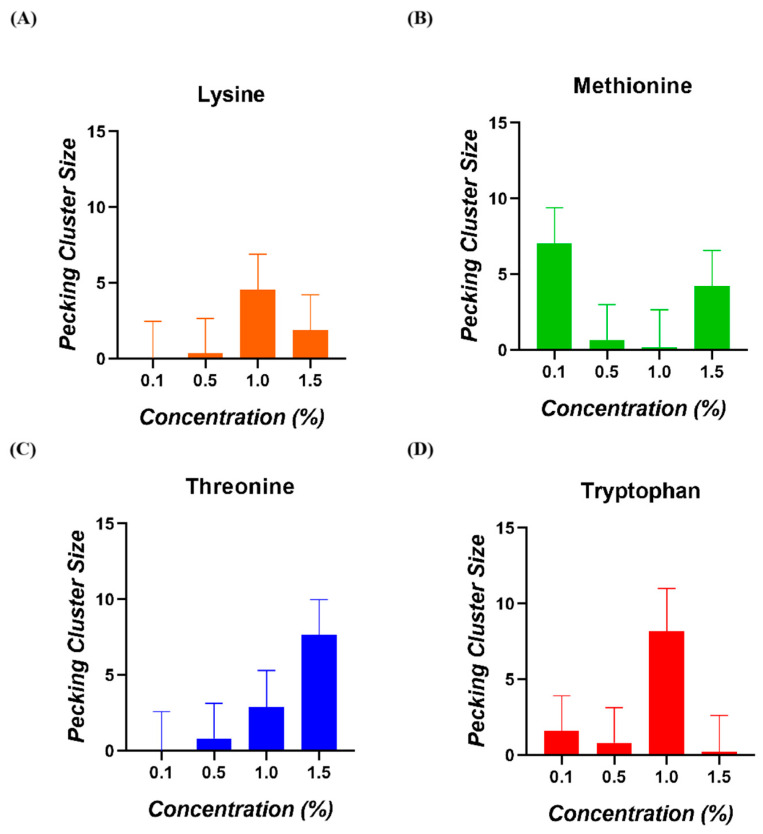
Pecking cluster size of broiler chickens for (**A**) Lysine, (**B**) Methionine, (**C**) Threonine, and (**D**) Tryptophan.

The ANOVA analysis indicated that the interaction between amino acid and concentration had no significant effect on the pecking cluster size of broiler chickens (*p* = 0.316). Furthermore, Tukey’s post hoc test did not show any significant differences among the four concentrations tested that influenced this parameter (Figure 5).

## 4. Discussion

Gustatory sensitivity can be determined through preference and SMI studies of taste-active compounds and the expression of detection thresholds. This concept is associated with the taste intensity of a compound that allows it to be perceived and distinguished [16] and that determines the ability of a species to detect and prefer it at a specific range, which may be favorable for a certain intake of food when reached [6]. The current study analyzed preference and consumption driven by sensory motivation for four essential amino acids, determining consumption behavior in broiler chickens that varied based on the concentration of the compounds administered. Preference tests are one of the most commonly used methods for estimating feeding behavior. However, they can often be misinterpreted as measures of the compounds’ palatability [9]. Like other species, analyzing consumption patterns better approximates animals’ hedonic responses to food or solution consumption. For this reason, we also examined acceptability and introduced a novel measure called pecking cluster size to describe palatability for water solutions.

The results obtained in this study did not reflect preference behaviors for the amino acids evaluated, with trends towards non-preference for Threonine and Tryptophan at a 1.5% concentration also noted. Previous studies on avian preferences identified aversions for other umami compounds such as MSG, inositol monophosphate (IMP), and other non-essential amino acids [1,2,6], which are in accordance with the findings of this assay if we consider that the amino acids studied are also categorized as umami compounds. On the other hand, the studies above also presented certain discrepancies when evaluating the synergistic potential of the MSG + IMP association on feed intake. This result cannot be discussed with the findings of this work as this parameter was not analyzed. Methodological differences applied by researchers could explain the differences in the obtained results.

An example of this is the type of compound delivery matrix used. For instance, Yoshida et al. (2015) used a solid feed matrix [6], unlike Cheled-Shoval et al. (2017) and, subsequently, Yoshida et al. (2018), who used water as the delivery matrix [1,2], similar to the current study. Water is a tasteless matrix that facilitates the precise administration of compounds and allows for an exact consumption measurement [2] without the influence of intrinsic flavors, which could occur with a feed matrix. The duration of the trials also influences the consumption parameters obtained in these tests. The current study administered the solutions for 4 h. Previous studies, such as Yoshida et al. (2018), conducted short-term tests with 5 min of the compound on offer [1], contrary to Cheled-Shoval et al. (2017), where the trials were 6 and 24 h [2]. This is relevant since post-ingestive processes intervene in feed consumption in response to the detection of nutrients such as amino acids and other chemical signals, and this regulates the secretion of intestinal hormones in the hunger and satiety cycle and intestinal motility in birds [5], which is a function that has also been previously described in mammals [17]. From a behavioral standpoint, it has been confirmed that short tests condition the animal to only consume the preferred food, reducing the possibility of consuming other compounds offered [9]. The fasting time prior to the start of the tests has also been studied as a determining factor in the results, given that its primary objective is to increase the consumption of the compounds offered. This study incorporated a fasting period of 1 h, unlike the application of prolonged fasts of 17 [6] and 24 h [1]. The background highlights the need to standardize the methodology used in this type of testing for broiler chickens. This standardization aims to reduce biases and achieve comparable results across studies. In this context, our previous research identified the optimal combinations of pairs of animals assessed with liquid matrices, which we applied in this experiment [7].

Regarding the analysis of SMI conducted in this study, a trend toward a lower SMI was observed in birds receiving 1.5% Threonine, which is consistent with what was seen in the preference analysis, where this amino acid showed a tendency toward non-preference at the same concentration. The results support the fact that the previous lower preference observed translates into a significantly lower consumption in absolute terms. Also, a trend toward a higher SMI was observed in birds receiving 1.5% Lysine, which supports the preference (non-significant) observed in the previous analysis. Taste sensitivity is related to the physiological activation of taste sensors in the buds in response to nutritional compounds in the oral cavities during food consumption. This premise has been demonstrated through studies on birds’ feeding behavior that have determined the presence of specific taste receptors, such as the T1R1 and T1R3 sensors associated with detecting umami compounds [18]. This corroborates that taste plays a vital role in food selection and possibly in the motivation of eating behavior [19] and that the taste sensations perceived by birds guide their nutritional choices through the sense of taste, which is a point of great relevance for animal nutrition [20,21]. On the other hand, it is known that birds can adjust feed consumption to compensate for states of nutritional deficit, such as amino acids deficiency [22], which would lead to alterations in feed consumption. A recent publication from our group examined the nutritional status of broiler chickens as a factor influencing feeding behavior. The study found that reducing crude protein and four essential amino acids in their diets negatively affected production parameters and increased their sensitivity to Lysine [23]. In the current study, we used commercial diets that fulfilled all the nutritional requirements of the birds. Future research should assess feeding behavior by evaluating acceptability and pecking cluster size in birds subjected to different nutritional conditions.

The differential acceptability of a compound is closely related to the regulation of voluntary feed intake, influenced by sensory, post-ingestive, and physiological factors [20,21,24,25]. Among these, sensory perception serves as the primary trigger, modulating the total intake of one amino acid relative to another. In the present study, birds exhibited higher acceptability for Methionine than Tryptophan. Taste preference assessments did not reveal significant differences in preference values across the tested concentrations of Methionine or Tryptophan. Nonetheless, a trend toward a lower preference for Tryptophan at the 1.5% concentration was observed. This greater acceptability for Methionine aligns with the preference data, considering that acceptability is defined based on the total intake of a single amino acid source. Consequently, Methionine appears to be more widely consumed and preferred to water. In contrast, Tryptophan exhibited a lower intake and a tendency toward reduced preferences.

Methionine is recognized as one of the main limiting amino acids in broiler diets [26], and its optimal inclusion has been extensively studied due to its critical role in protein synthesis and other essential metabolic functions [27,28,29,30]. Tryptophan, in turn, is also considered a limiting amino acid in poultry nutrition, essential not only for its optimal productive performance but also for animal welfare, as it serves as a precursor to serotonin [31,32]. Adequate levels of dietary Methionine have been shown to improve feed utilization efficiency by enhancing protein synthesis and metabolic processes. This improved nutrient utilization can indirectly influence appetite by reducing feed intake due to better nutrient satisfaction or stimulating intake when Methionine deficiency prompts compensatory feeding behavior [33,34]. Conversely, inadequate levels of Tryptophan may reduce feed intake [35]. However, these amino acids’ sensory and post-ingestive effects at different concentrations remain insufficiently elucidated in feeding behavior trials. Concerning the effects of compound concentrations, the results from this study were inconclusive. Higher total intakes were observed at the lowest (0.1%) and highest (1.5%) concentrations, suggesting a potentially non-linear response. Therefore, further research is warranted to understand how varying concentrations of these amino acids influence birds’ feeding behaviors and intake regulation.

Regarding the interaction between amino acid type and concentration on acceptability, a higher Lysine intake was observed at the highest tested concentration (1.5%) compared to lower concentrations (0.5% and 1%). This may reflect birds’ efforts to meet optimal Lysine requirements for adequate productive performance. Lysine is a limiting amino acid in poultry nutrition that directly influences carcass development [36]. Several studies evaluating optimal Lysine inclusion in poultry diets have reported that concentrations of 1.07% and 1.09% maximize weight gain, carcass yield, and economic returns [37]. Additionally, elevated dietary Lysine levels during early growth stages have been associated with increased muscle accretion, ultimately improving final body weight and positively impacting gastrointestinal development, enhancing nutrient absorption and feed conversion efficiency [38,39]. A higher intake was observed at the lowest evaluated concentration (0.1%) compared to the 1% level for Methionine. This finding is consistent with previous reports indicating that excessive Methionine supplementation may negatively affect feed intake and growth performance [40]. These findings suggest that birds modulate their feeding behavior according to their physiological needs, likely aiming to optimize growth and overall performance.

In this study, we introduced pecking cluster size as a new way to approximate the palatability of amino acids. This consumption pattern was measured by calculating the ratio of the number of pecks to the number of bouts for each animal. We did not find any significant differences among the various concentrations tested. For this assay, the animals were fasted for one hour, and the amino acid solution was available for four hours, with the first ten minutes being recorded. However, during many of these recordings, we observed low activity levels with the provided solutions, resulting in several pecking cluster size values close to zero. Moreover, in chickens, in contrast to rats, each peck may deliver different amounts of solutions. Therefore, extending the recording period or differencing the different kinds of pecking may be an important consideration for future research.

## 5. Conclusions

Amino acid solutions such as Threonine and Tryptophan tended to show less preference at the highest exposed concentrations (1.5%) concerning drinking water in growing broiler chickens. This was confirmed in the case of Threonine when performing the SMI analysis, which presented a lower consumption in absolute terms than water when exposed simultaneously. The opposite situation occurred with Lysine (1.5%), which numerically showed a greater preference ratified by a tendency toward a higher intake than drinking water through the SMI analysis and a higher intake when it was presented alone during an acceptability test concerning other concentrations of the same amino acid. No significant differences across amino acid concentrations were observed when palatability was measured with pecking cluster size. This lack of differentiation may be attributed to short recording periods, low activity levels noticed during the initial minutes of testing, and differences in solution intake between species, where chickens in each pecking cluster may drink different amounts of the solution. The trends and non-significant results also reflect a significant data variability, reinforcing that it is necessary to standardize feeding behavior tests in birds according to their feeding patterns and nutritional needs. The amino acids examined in this study are recognized as limiting factors in poultry nutrition, making their inclusion vital for the growth and development of broiler birds. In practical terms, understanding the interaction between preference, SMI, acceptability, and palatability that determine birds’ taste perception of amino acids at different concentrations is crucial for developing effective feeding strategies that improve poultry diets and increase production efficiency.

## Data Availability

The data are available upon reasonable request to the submitting author.

## Supplementary Materials

The following supporting information can be downloaded at https://www.mdpi.com/article/10.3390/ani15111574/s1. Table S1: Composition and chemical analysis of the starter diet used in the experiment.
