# Supplementary material for: Taste Preferences in Broilers: Behavioral Evaluation for Varying Concentrations of Four Essential Amino Acids
Source: Animals (Basel). 2025 May 28;15(11):1574. doi: 10.3390/ani15111574 (PMC12153537; doi:10.3390/ani15111574)
Supplement: Supplementary file 1 [file animals-15-01574-s001.zip › animals-3645303-supplementary.pdf]

animals

MDPI

Supplementary Table S1. Composition and chemical analysis of the starter diet used in the experiment.

Item	Starter
Ingredients (g/kg)	
Corn	554.2
Soybean meal (47% protein)	270.3
Ground wheat	50.0
Rapeseed meal	40.0
Gluten meal (60% protein)	30.0
Olein oil	16.5
$CaCO_3$	14.4
$CaHPO_4$	11.8
NaCl	4.4
Lys	2.2
Met	2.1
Micofix plus[1]	0.005
Coccidiostat	0.5
Multivitamins-mineral-phytase[2]	2.0
Formicit dry[3]	1.0
Analyzed nutrient composition (%)	
Dry matter	88.9
Crude protein	22.9
Crude fiber	3.4
Ether extract	3.8
NNE	53.7
Ash	5.1
Estimated nutrient composition	
Metabolizable energy (Kcal/kg)	3050
Ca (%)	1.05
Available P (%)	0.47

[1]Integral solution for mycotoxins (Virbac Centrovet, Santiago, Chile); [2]Contains per kilo of premix: 8000 UI of Vit. A, 2500 UI of Vit. D3, 15 UI of Vit. E, 1.5 mg of K3, 1.5 mg of Vit. B1, 5 mg of Vit. B2, 35 mg of niacin, 13.1 mg of calcium pantothenate, 2.49 mg of Vit. B6, 0.012 mg of Vit. B12, 1 mg of folic acid, 0.1 mg of biotin, 399.5 mg of choline, 25 mg of Fe, 70 mg of Mn, 60 mg of Zn, 6 mg of Cu, 0.15 mg of Se, 100 mg of antox, 0.5 mg of I,100 mg of Hostazym X, 50 mg of Optiphos G; [3]Preservative anti-salmonella sp. For food preservation (Veterquímica SA, Santiago, Chile).
